# Odontological Society of Pennsylvania

**Published:** 1889-06

**Authors:** 


### Reports of Society Meetings.

### ODONTOLOGICAL SOCIETY OF PENNSYLVANIA.

THE REGULAR MEETING OF THE ODONTOLOGICAL SOCIETY OF PENNSYLVANIA, WAS HELD SATURDAY EVENING, MARCH 2, 1889, IN THE ROOMS, ARCH AND THIRTEENTH STREETS.

President Kirk in the chair.

### DISCUSSION OF DR. PEIRCE'S PAPER.

*Dr. Sudduth*—I have listened to the paper with much pleasure but shall have to take exception to several statements of the essayist in regard to caries. The first is in regard to the recuperative power of the teeth. I think there is no question in the minds of active workers, that the tooth is a passive agent in the process of decay, and that the only resistance offered by it, finds its expression in the pulp cavity. The office of the pulp is that of a formative organ, and when irritation, either by abrasion or chemical disintegration, occurs to the distal ends of the fibres, the irritation is transmitted to the odontoblastic layer, and as a result of such irritation, we have secondary dentine developed; which, so far as we have been able to demonstrate, is the only resistive action shown by the tooth in the process of decay.

I use the term decay as opposed to the term caries. In my experience I have never seen decay of cementum in the same manner as we have decay occurring in the enamel and dentine of the exposed portion of the teeth. The breaking down of the cemental portion, or root of the tooth, is analogous to caries in bone, and is carried on by the same process, viz., Osteoclasts. We should distinguish between the terms caries and decay—decay, for that process which occurs in the exposed portion of the tooth, and caries for the roots of the teeth, and especially for the breaking down of the bony tissue. I have never seen decay occuring in the cementum. In cases of pockets forming around the teeth in pyorrhea alveolaris, it has been my experience, that there is almost never a breaking down of the roots of the tooth, in the same manner that we find in the crown of the tooth. That absence from destruction has been a marked feature in influencing my deduction, in regard to the difference between the two processes. Dr. Peirce also spoke in regard to decay's being more active in youth than in advanced age. There are two or three clinical phases of the question, which I have not been able to settle in my own mind. I have not had the same experience in years of practice that many of you have had, and consequently I do not feel justified in saying anything definitely in regard to this one point, yet I would offer this suggestion in regard to it, that if such is the case that caries is more prevalent in youth than in mature life, the solution to the question will be found in that these teeth, being erupted in a poorly calcified condition, are broken down by the first result of the fermentative process, and therefore pass out of the way first—we have remaining only the strong teeth in after life, whereas, the weak teeth have disappeared in youth.

It seems to me that we know sufficiently regarding the process of decay to say positively that decay does not occur through the general acid condition of the saliva. Erosion may be the result of that condition, but decay, as we know it, which is the breaking down of tooth structure first, by decalcification, and secondly, by the disintegrating process that takes out the basis substance, is due to something else than the acid condition of the saliva, and these old acid and other theories must be done away with, and give place to later ones.

Another point I noticed made by Dr. Peirce, was that of the periodicity of decay; during certain times of life, in youth or middle life we have a greater amount of decay than at other times. That an overtaxed condition of the system, has anything to do with the condition of the tooth itself as a resisting agent in the matter, I cannot admit for a minute, yet periodicity may be due to a certain action, which we have not yet fathomed. I have noticed it in my own case; but why it occurs I cannot say. It may be that at these times the patient gives less care to the teeth than at other times.

The, so called, exciting cause, abnormal conditions in secretions, mentioned by the essayist, has been answered in the statement that the secretions themselves, except in a very few instances, can not be said to give rise to the breaking down of tooth structure. You may have erosion at the gingival margin, but that is not decay. It is a different kind of process, and should come under the class of erosion rather than decay. We are positive that acids will not cause decay in teeth. They break down enamel and dissolve out the lime salts of the teeth; but the form of the den tine will remain the same. We must have something else besides acids. We must have a digestive ferment, and the only factors that will develop it are the micro-organisms found in the oral cavity. The germs are the exciting causes; others things are only accessory.

A perfectly sound isolated tooth, without a break in its continuity, will never decay. Micro-organisms cannot enter the tubuli unless the dentine is first decalcified. It is only when decalcification has taken place that they can enter and find a place to work. They must be in an enclosed cavity, and that is why we do not have decay in the pockets that form around the roots of teeth in pyorrhea alveolaris; the saliva enters freely and dilutes the acids produced by the micro-organisms at that point. The predisposing causes are, first, malformations with poorly calcified sulci; second, irregularities which give rise to pockets where micro-organisms can collect; and, third, abrasion, which is seldom a predisposing cause. The exciting cause in decay, which I would confine to the crowns or the exposed portion of the teeth is, beyond question, microbes, and the predisposing causes are malformations.

The paper was an excellent one, and the points to which attention was called, regarding the conditions which predispose to poorly developed teeth, were well taken. It was a presentment we can all learn from, and the only exception I take, is in regard to the active agents in the causation of decay, and the distinction between caries and decay, and that the tooth is a passive and not an active agent in the process.

*Dr. Faught*—I was very much pleased with the paper and with the practical aspect of it. I think it sets forth some of the causes of decay. I emphasize the expression practical, because we all recognize the periodicity of decay, tendency and heredity are undoubtedly factors in certain conditions of the system other than the teeth, and must undoubtedly be considered in the question of combating caries. If caries be the result of acids breaking down the enamel, or of germs entering and disintegrating the dentine, then after we have placed the teeth in proper hygienic condition, we might be assured of the permanency of our work. But such is not the case, for with our patients constantly under our care, seeing them at near intervals, knowing them to be cleanly and to use the proper means to secure this end, even then we meet with recurrent caries; and whether it is a factor or not, we know of concomitant lowered nerve tone in the patient, and of which we believe the fluctuations make constant demands upon us for renewed effort to protect the teeth. This fact I want to emphasize, for I believe that one of our worst enemies is the nervous influences on decay. When lowered, caries progress rapidly and is of frequent occurrence; when normal, we find the teeth do not need so much care.

*Dr. Sudduth*—I would like to ask Dr. Faught a word in regard to lowered nerve tone. Might it not be that patients, owing to this condition, come to us with their mouths in a less cleanly state?

*Dr. Faught*—I think not. I try to impress upon my patients the necessity of oral hygiene. I take entire charge of their mouths, sending for them when I deem they need attention. They are patients who are very careful in all matters of cleanliness. They would not neglect it on account of lowered nervous tone. I find it invariably the case that in many instances we can trace the progress of caries and fix it upon nervous conditions. I speak of one lady in particular who passed through great trouble and her teeth began immediately to break down. She then had new conditions of life put upon her, and I saw that the teeth responded. Seeing so many of these cases which I care for, I must think that the question of nerve tone or nutrition in some way has decided influence upon dental caries.

*Dr. James Truman*—This is well-ploughed ground; but I am pleased that Prof. Peirce has responded to the request for a paper in the way he has. It is always satisfactory to get what I call an original idea, and I think I have received one to-night from the paper read, and that is: That, at the period of childhood, there is a deficiency of lime salts to meet all the requirements of the system; and that therefore decay progresses more rapidly. While it is true that teeth at this early age lack density, I question whether it is from a want of the constituent elements in the organization to supply them. That this can have more than an indirect influence may be questioned; the direct being in the tendency to fermentation and acid products at that age, there being necessarily an increased tendency in this direction at this time. I think he made the statement that teeth are not destroyed, if I understand him correctly, by the secretions of the follicles.

*Dr. Peirce*—When there is tartar deposit in the mouth.

*Dr. Truman*—Then you accept glandular action in the matter of erosion?

*Dr. Peirce*—Yes.

*Dr. Truman*—There are two prominent points in the paper upon which I wish to enlarge. I cannot agree with Dr. Sudduth that the density of teeth has nothing to do with the action of caries. The condition of the teeth necessarily affects the progress of caries, the density acting as a constant barrier to the inroads of the disease.

*Dr. Sudduth*—Allow me to correct, if you please. I said that there were no lime salts thrown out except upon the surface of the pulp, and that is what protects it from the influence of decay.

*Dr. Truman*—I also take exception to that. In irritations that do not extend beyond certain limits, there is increased deposition of lime salts.

*Dr. Sudduth*—In the pulp cavity only.

*Dr. Truman*—You will find it in the tubes of the teeth. I am well aware that I am in opposition to many—Black, Wedl and even Tomes—on this subject. Nevertheless, my convictions are positive, that depositions occur in the tubuli of the teeth, and that in sufficient quantity to act decidedly against the progress of caries. These gentlemen will probably not deny that the fibres that penetrate the tubuli are prolongations of the pulp, and are hence part of that organ and subject to its conditions and its physiological changes. If, therefore, secondary formations are possible in the main organ, independent of the odontoblastic layer, they are possible in the minutest branch. There can be no theoretical argument that will apply to one that will not apply to the other; hence I regard the transparent zone of Tomes as a possibility. Any other view of the subject seems to me to be antagonistic to fact and reasonable analogy. Dr. Sudduth made the remark, if I understood him, that there is no destruction without micro-organisms; that the secretions of the mouth never produce destruction.

*Dr. Sudduth*—Acids produce destruction on the free surfaces of the teeth—erosion—but not decay.

*Dr. Truman*—Then I have no quarrel with you on that point.

*Dr. Sudduth*—I have here, to-night, a section of ivory, in which a ball was imbedded, and in which this translucent zone shows very prettily between the cavity and the sound dentine. I would say in regard to this translucent zone, that I have taken sections of teeth where this transparent zone showed as prettily as it does in in this section, and reproduced the normal appearance by soaking them in ether. I have also taken the sections of teeth and put them in an imbedding mass of paraffin, melted, and produced this transparent zone, getting the same appearance. Dr. Miller admits that there is a possibility of error; yet the fact that we can take specimens, and by dissolving out by the use of ether or essential oils the fatty portions of the fiber, which I have no doubt are dissolved out, thus clearing up this translucent zone proves, to my mind at least, that this translucent zone is a product of the disintegration of the fibrils themselves, which always goes ahead of the process of decay. I have never been able to demonstate that there is any decalfication there.

*Dr. Kirk*—Before the subject is closed, I would like to ask a few questions. Dr. Peirce used the term, "recuperative power of the teeth." As I understand it, Dr. Peirce is talking about one thing and Dr. Sudduth seems to be talking of something else. Dr. Sudduth confines recuperative action to the pulp cavity. Dr. Peirce's term applies to increase of density in teeth, from a lower condition to one of greater density. I think the microscope is not the only means we have at our command of distinguishing this question. There are certain facts that, to my mind, the microscope has not explained.

*Dr. Sudduth*—In what portion of the tooth do you say this occurs; in the enamel or the dentine?

*Dr. Kirk*—In the dentine. I read a paper in 1881, called "The Tooth in Pregnancy." In this I noted the various stages of dentine while women were in this condition, finding that when they bore children there was a decided loss of density of tooth structure, but in which repair afterwards took place.

A patient came to me from the hands of a dentist who had the previous year inserted thirty fillings. That lady has been a patient of mine for eight or nine years, and in that time I have not inserted a half dozen fillings. It is not due to difference of ability, because I know the man who filled the teeth to be a good operator, and I attribute the difference in the quality of her teeth simply to restored condition of health, and I know her teeth to-day are harder and denser than when I first saw her.

Regarding this matter of density I hold that a man whose fingers are educated, can tell the difference, and that his observation is just as good as though the microscope were used, whether he is cutting cheese or marble ; and we have seen the periodicity of decay, with increase and decrease of density in tooth structure too often to be misled. What it may have to do with the question of the osteo-dentine, or secondary dentine, I do not know or care; but I wish to go on record as believing that tooth structure, which I will limit to dentine for the sake of argument, although I am not prepared to say it does not effect enamel—gets harder and stronger.

Another point. I do not quite understand the difference Dr. Sudduth makes between the terms decay and caries. It seems to me that they are perfectly convertible terms. He said that acids were not capable of causing decay of the teeth. He further says that all the acid does is to make a roughing of the enamel, which is followed up by the ingress of micro-organisms in that condition, which act as a fermentable substance, and he further states that fermentative action is the production of the microbes. It therefore seems to me that it is a localized action—localized because the micro-organisms are produced in one spot. They simply remove the lime salts from the tooth, and the gelatinous, or organic matter left becomes broken down. That is as I understand it. In regard to this acidity which we observe in the mouths of children, and where erosion of the teeth take place at the neck, Dr. Peirce's idea does not explain it all, I think. It explains the relations of the teeth to an acid condition, but does not afford an explanation of why the acid condition exists. I believe it does exist in the period of childhood much more than it does later on—part from a lack of cleanliness, and my own explanation as to its cause, which is purely theoretical, is this: Where the tissues are rapidly growing—where any of the structures are rapidly growing, means an increased determination of blood to that part, and a condition of mucous membrane the same as we see brought about by a local vaso-motor disturbance. The effect on the mucous membrane at that time, is to produce an acid condition. The teeth, of course, being soft, and of a looser texture at that time are more rapidly acted upon.

I would like to ask Dr. Sudduth whether the teeth do not become denser through various periods of life, or having become dense, may not become less dense?

*Dr. Sudduth*—That goes into a question of chemistry, and one I think our president should himself answer. What we observe in the matter of density is the enamel, not dentine. The exposed covering of the teeth in the mouth is entirely enamel and the variations in density and softness relate more particularly to the enamel than to the dentine, and that is one reason why I asked the question of Dr. Kirk, whether it was the enamel or the dentine. We know that fermentation is a cause of decay, not a sequence. You cannot break down the substance of a tooth with an acid, You can decalcify bone, but you still have the form of the bone. The acid has simply taken out the lime salts. Caries is a process occurring in organic tissue in which the tissue itself plays an active part. Caries is confined to tissues that are buried in the soft tissues; whereas, decay occurs in the exposed portion, and is a process which originates outside the tooth itself, and in which the tooth plays a passive part. Decay is the result of microbic action. The free acid in the mouth causes erosion; but does not destroy the form. Decay destroys the form and is a process that occurs in organic tissue.

*Dr. Kirk*—I would like to ask if Dr. Sudduth believes that a tooth when once erupted remains the same all through life?

*Dr. Sudduth*—Certainly not. Calcification of the dentine at the time of eruption is not complete. It is only after a considerable time that the tooth becomes thorougly calcified. The enamel after the tooth is erupted, however, can change its density only from the saliva. When the tooth erupts the enamel is forever shut off from its original source of supply of lime salts, and the formative organ, the ameloblastic layer, becomes hornified and loses its function. Any change which takes place thereafter in the enamel is a chemical and not a vital action. The source of lime is then from the saliva and if there is any increase in the chemical constituency of the tissue, it is from without and not within.

*Dr. Faught*—I fear that my remarks have been misunderstood, they simply had this reference: If acids alone destroyed the tooth we should have comparatively good results as dentists in caring for the teeth. I do not believe this to be true, and even if we understood this matter thoroughly, I still believe we should have a world of trouble in preserving the teeth, as a result of conditions existing in the tooth and from the lowered nerve tone. I am not prepared to say how much is due to the one or to the other, but I believe clinically, that decay goes on and gives trouble on account of the lowered tone. If we only had the influences mentioned by Dr. Sudduth we should have a good time caring for the teeth. I should like to believe that there is more or less fluctuation in the conditions of the enamel as well as the dentine.

*Dr. C. N. Peirce*—Dr. Sudduth instances a case of Pyorrhœa where the pockets are large and open, and, though the cementum on the roots of such teeth is constantly subjected to the influences of the saliva, yet he has never observed decay attacking the cementum of such roots. I should be obliged to Dr. Sudduth, or indeed to anyone, for a view of a case of progressing decay where calcic pyorrhœa is present. I have never seen dental caries or decay progressing, in any dental tissue, where pyorrhœa alveolaris was present. Therefore, the fact that cementum has not, under such circumstances decayed, is no reason for assuming that it will not, under more favorable conditions, become the seat of this disease.

*Dr. Sudduth*—Has Dr. Pierce seen decay in cementum?

*Dr. Peirce*—Yes. If Dr. Sudduth would like an illustration of cemental decay, I would refer him to the mesial or distal surfaces of bicuspids, or molars, where a partial set of teeth on rubber is worn, with the plate impinging upon these surfaces, so as to retain particles of food, or any fermentable substance, in contact therewith. I think, in the large majority of such cases, cemental decomposition will begin within six months after the insertion of such partial dentures.

*Dr. Sudduth*—The cement is so thin at that point that it could hardly be called decay, no pocket is formed in the cement.

*Dr. Peirce*—Regarding the resisting power of the tooth, my experience has been that it increases or diminishes with the modification of certain systemic conditions. It certainly is the result of observation, that teeth for months, and even years, have been gradually decomposing, and then through the influence of systemic change, decay entirely ceases—this having been preceded by a change in the oral secretions, which is also the result of constitutional or systemic conditions.

This retardation, or as is somtimes observed, the entire or complete arrest of dental decay, followed by a brown eburnated surface where the decomposition had previously been active is due, I should judge, to both local and constitutional conditions, and may very properly be termed a vital, as well as a recuperative effort of the tooth, for the pulp has been largely instrumental in its accomplishment. That the tooth, under ordinary circumstances, increases in density, I am well persuaded, and that this is accompanied by an increased resistive power, is also well attested.

The discrimination Dr. Sudduth makes between dental caries and dental decay, I think quite pertinent, and will endeavor to conform to it in my subsequent remarks.

In speaking of the origin of decay, I intended to be explicit in my paper, and give due and full credit to the organisms in their productions. I think I said emphatically, that dental decay is the result of the presence of low forms of life, but before they can take hold of the dental tissue, and act upon it as a digestible ferment, there must be a pabulum for their habitat and multiplication. There must be a decalcification so that the organic matter remaining shall offer the needed elements for home and reproduction ; more than this they may do, some varieties of them may by their action consume the sugar in the debris, and secretions, and excrete an acid, and this may contribute to the primary decalcification, but this result may be modified, or even wholly arrested by the influence of systemic conditions upon the oral secretions.

The statement made in the paper regarding the deficiency and subsequently the excess of lime salts being dependent upon the demands of the system, varying with youth and advancing years, is in the form of an inquiry. But the invariable change taking place in the condition of the teeth with youth, from youth to adult life, is a strong argument in favor of the proposition. And, again, the noticeable deterioration of the teeth during periods of gestation and lactation, and their improvement after these periods have terminated, is also an argument in favor of the influence of systemic demands. These changes are observed quite irrespective of the effort of cleanliness.

*Dr. Kirk*—Is that due to the altered secretions of the mouth or to a change in the structure of the teeth?

*Dr. Peirce*—To both; the secretions have changed because the supply and demand of nutrition has changed. The blood, at one time impoverished in certain elements, is at another period surcharged with the same elements ; and through this same nutritive fluid, it is my conviction, the teeth are also increased in density with adult life and advancing years. The continuity existing between the organic and inorganic is modified, that is, increased or deteriorated.

*Dr. Sudduth*—In which, the enamel or the dentine?

*Dr. Peirce*—In the dentine especially is it observed. There is so little organic matter in the enamel that it is not so marked, but I believe it is also proportionately increased in density. Only a few days since an illustration of this varying density and want of continuity was experienced, in the effort to excavate a cavity in a tooth which had on a previous occasion, been very dense, but on a recent effort it offered little more resistance than would a piece of cartilage.

*Dr. Sudduth*—Was that in the enamel or the dentine?

*Dr. Pierce*—It was especially in the dentine, although the enamel offered much less than the usual resistance. I have frequently noticed the difference in the structure and density of the same teeth in different periods of life and in different conditions of health—and this notwithstanding that Drs. Sudduth, Black and others say there is no change. The sharp excavator tells the story.

*Dr. James Truman*—Microscopists are remarkably apt to be dogmatical. Dr. Sudduth seems to be no exception to this rule. He asserts to-night that the investigations of Rainey demonstrated the existence of calco-globulin in the teeth. He does not give us any confirmation of this statement—beyond mere assertion.

*Dr. Sudduth*—I have often made the experiment myself.

*Dr. Truman*—You have not explained how or when. You also assert that the enamel does not increase in density after its first formation. I presume there is not a dentist in this or any other country that does not know from practical observation that it does change.

*Dr. Sudduth*—You have misunderstood me. I said that if it did become denser it was from the effect of the saliva.

*Dr. Truman*—Here we have another assertion. If it becomes denser, it must be by imbibition of fluids from the interior. You assert that there is no passage of fluids into the enamel from this direction. I must say dogmatically, that between every enamel rod there is something. What is it? You call it calco-globulin. I say it is organic tissue, and I have the investigations of Bödecker and Heitzman to support this assertion. Aside from this there are certain physiological effects produced, not possible if this were not so. The difficulty of making examinations in this tissue will, probably, prevent anything positive's being said in regard to this. Of one thing we may be certain—that old age brings with it increased density in tooth structure.

*Dr. Sudduth*—Is it the dentine or the enamel?

*Dr. Truman*—In both. If the physiological process of nutrition failed, there would be no teeth in the mouth. The density increases as age advances and the enamel, with the other tissues, has its source of nutrition. You say that dentine is formed altogether by the odontoblastic layer at the surface. You acknowledge that the density of dentine is increased. If the formation is confined to the superficial layer how is this accomplished? How are the lime salts deposited in the dentine? Is it by the action of the fibres? Or is it simply by imbibition? I contend that the only explanation is through the action of the fibres. It is impossible to prove this, and yet we are familiar with conditions that seem to demonstrate it. It is not unusual to find so-called senile dentine with the tubes partially or wholly obliterated; and there is another state, analogous to this, which may occur in middle life, a condition I have never yet seen explained, or even alluded to, except by myself. It is in cases of early loss of teeth, not by pericementitis or pyorrhœa alveolaris, but through a condition of the tubes in which absolute calcification has apparently taken place, in other words, the tubes with their parietes have been wholly obliterated. This prevents nutrition, and the teeth are slowly but surely thrown out of the mouth as foreign bodies. A case of this kind came to me some years ago. The gentleman was about 35 years of age. He possessed a beautiful set of extremely dense teeth. They were gradually loosening and without pathological conditions. I exhausted my skill in efforts to save them, and he eventually lost every one, and that in a comparatively short period. Examination microscopically demonstrated the cause. The tubes were entirely obliterated except for a short distance near the remains of the canal. The dentine in sections presented a clear transparent appearance, as devoid of lines as a piece of glass.

*Dr. Sudduth*—How do you know they were not always that way?

*Dr. Truman*—Had they been that way originally, nutrition would have been impossible in the earlier as it evidently was at a later period. Did you ever see a tooth without tubes?

*Dr. Sudduth*—I have never met with teeth in which the tubes were absent.

*Dr. Truman*—You will have to take my word for it, as I have not the specimen to show, that these tubes were filled up and obliterated by depositions of lime salts.

I do not believe in "periods of destruction." I cannot understand that there are times when the individual will lose more teeth by caries than at others. This loss may occur at any age through physiological disturbance resulting in lowered nerve tone and increased acid secretion.

*Dr. W. G. A. Bonwill*—I wish to give you an illustration of the change in the dentistry of teeth. A gentleman 34 years of age came to me a while ago; he had good dense teeth, but they were loosening one by one. He was sent to me by two others, whose teeth I had treated, being similar cases. He had been to four of the very best dentists in the city within the previous three months. They told him he would lose them all. He went to Prof. Agnew, who said he must lose them. He went to Garrettson, DaCosta and Pepper, who all said the same thing. I found that all that was needed was a little common sense treatment. He had used a tooth brush most unmercifully, and the tissue was torn away. You could see the bone bright and clean by passing up where neither the brush nor the pick has ever touched, decay had gone on. To effect the cure I simply cleansed the teeth and cut off all super-abundance of tissue. I never saw teeth harder in my life. The gum has nearly grown around the teeth, and there is as perfect a condition as before. The roots of the teeth were as solid as the enamel itself.

*Dr. Tees*—My attention has been lately called to several instances of the beneficial effect of lime water, upon both the temporary and the permanent teeth of children under my charge. On account of the way in which children are fed in this country, being given all manner of food from earliest infancy, we seldom meet with such cases. The first case was that of a young girl about fourteen years of age. She had a most perfect set of twenty-eight teeth, with no decay in any of them. I asked the mother what she fed her upon in childhood. She replied, that at birth nothing would lie upon her stomach. The physician directed the child to be fed upon cow's milk with a tablespoon of lime-water in each tumblerful. Upon this diet she was brought up until she was five years of age. After that she ate bread and butter and eating sparingly of other articles of food, and refusing to eat candies, cakes and other goodies which children generally crave. This young lady is now eighteen years of age, and has one cavity filled. I am sorry to say that she has affected the trashy food of American young people for the past two or three years. I asked the mother how she accounted for the decayed condition of her son's teeth. She replied, "Doctor, his grandmother brought him up."

In another instance a boy five years of age had perfect deciduous teeth, while his sister, three years of age, had teeth in an advanced state of decay. The mother said that the boy had been fed upon limewater and milk until he was four years of age, and she personally saw that it was given him; while the little girl, during babyhood, was in charge of the nurse, the mother's time being devoted to her invalid husband.

The following letter was received from Dr. E. Payson Quick, corresponding member:

SANTA BARBARA, CAL., Dec. 10, 1888.

*To the Recording Secretary of the Odontological Society of Penna.*

DEAR DOCTOR.—My health being somewhat restored, I opened an office in Santa Barbara, the first of November. I have a case on hand that I would ask the advice of the Society upon.

Patient is a lady, twenty-eight years of age, with good family history. Trouble began about twelve years ago, when a crackling noise was noticed upon opening and closing the mouth; This was followed by a gradual stiffening of the muscles and ligaments of the jaw, so that the mouth could not be opened more than half its normal width. At the same time the lateral movements became very imperfect. At times there is a twitching of the buccal muscles and a peculiar sensation in the tongue. There has been some, so-called, neuralgic pains, but not very severe. All the teeth are in place except the wisdom teeth, and quite a number are filled. The superior central incisors have had chronic abscesses for several years. I have treated them, and the teeth are now in a healthy condition. I have probed carefully for the wisdom teeth, but cannot detect them.

Now, is not this most likely a case of impacted wisdom teeth pressing upon the third division of the fifth pair of nerves, and by this constant irritation for years, bringing on this abnormal condition?

The patient has been in the hands of a number of physicians, but has not received any relief. I have advised the extraction of the second molars, but she fears the operation. So I am trying stretching the muscles and ligaments by placing a cork between the teeth for a few minutes each day, and also having her apply friction with the hands over the parts. By this treatment I have gained over half an inch.

What would the members of the Society advise in this case? Would they advise the extraction of the second molars? An opinion will greatly oblige,

E. PAYSON QUICK, D.D.S.

The following committee was appointed to answer the letter: Drs. James Truman, C. N. Peirce, S. H. Guilford, I. S. Fogg, S. E. Gilbert.

3249 Chestnut Street,
Philadelphia, Feb. 10th, 1889.

Dr. E. Payson Quick,

My dear Doctor.

Your communication of Dec. 10th was referred to a committee to consider and reply to. As chairman of that committee permit me to say that in my opinion, the case does not present all the indications of an impacted tooth, certainly not of pressure upon the inferior dental nerve. It possibly may have an indirect influence. Direct pressure, in the horizontal presentation, will produce severe neuroses. I doubt whether extraction of the second molar would avail anything. Loss of power in the muscles of the jaw is by no means unfrequent. In a similar case I used pressure between the teeth without much result. I then applied the galvanic current, with one pole at the neck with a wet sponge, and holding the other in my left hand. I used my right to manipulate the jaw, thus passing the current through my own body, modifying it to that extent. I had the pleasure of restoring the lady to a normal condition in about a week. Similar treatment might be of advantage, and the sooner the separation is accomplished the better.

Dr. C. N. Peirce does not think the conditions described warrant the extraction of the second molars, and before resorting to that treatment, with the present developments, would certainly advise application of the galvanic current.

Dr. S. H. Guilford is inclined to favor the theory of impaction of the third molars, as the cause of the trouble. He would recommend, first, the application of the galvanic current, and if that should fail, then the extraction of the second molars.

Dr. I. S. Fogg inclines to the opinion that an impacted tooth is the cause of the trouble. He would not however, resort to extraction before adopting the treatment recommended by myself, particularly in view of the slight improvement you report in your letter as having been gained by friction, etc.

Dr. Gilbert in an interview coincided with my views of the case.

Truly yours,
James Truman,
Chairman.

James Truman,
C. N. Peirce,
S. H. Guilford,
I. S. Fogg,
S. E. Gilbert,
} Committee.

### ODONTOLOGICAL SOCIETY OF PENNSYLVANIA.

The regular meeting of the Odontological Society of Pennsylvania was held Saturday evening, February 2d, 1889, at the hall, Thirteenth and Arch streets.

President Kirk in the chair.

### DISCUSSION OF DR. OTTOLENGUI'S PAPER ON IMPLANTATION.

*Dr. James Truman*—I am much pleased with the course that Dr. Ottolengui has taken in treating the subject, because I think it is high time that the dangers in performing this operation should be considered. In the cases illustrated, he shows distinctly how necessary it is to be careful. In some of the operations I have witnessed there has been a degree of carelessness exhibited, and I have contended that sooner or later there would be trouble caused by penetrating the antrum. I am only an observer, never having implanted a tooth; therefore I cannot discuss the subject. Dr. Kirk has had a large experience in implantation, and we should be pleased to hear from him.

*Dr. E. C. Kirk*—I am glad to have an opportunity to say a word upon implantation, particularly after such a good paper as has been given us. There can be no doubt that precaution should be observed from the beginning of an operation. I have observed a course somewhat different from the one described in the paper, because, I have relied exclusively upon bichloride of mercury as a disinfectant for instruments, socket, hands, etc. The case which he presents is very interesting. Some think that the mere penetration of the antrum will produce great inflammation. I do not see why it cannot be entered without so much danger. The case cited to-night is peculiar in itself. In the first place, he had a patient of marked hemorrhagic diathesis. In the second place, he not only opened the antrum, but injured an arteriole. The antrum was full of blood. Following this he closed the opening, and yet he maintains that he kept up antiseptic treatment. The history of the case proves, that no matter how well directed his efforts may have been, he did not succeed in it, because he evidently had a case of septicæmia, followed with purulent discharge. He could not thoroughly inject into the antrum and wash it out, because he was afraid of increasing the pain and causing a return of the hemorrhage.

I have been anxious to find just such a case, because we who believe in the operation are constantly having practitioners predict trouble. Dr. Ottolengui has shown one case where we may have difficulty—in a patient of hemorrhagic diathesis. After rupturing the arteriole and filling up the antrum with blood, to put in the tooth, and thus allow putrefaction to take place, was not good practice. In using a spear-pointed drill, I carry it with extreme caution to the depth I want the root. In drilling slowly, suppose you do make a perforation, any dentist can tell in opening any cavity when he runs into the pulp chamber. The cut of a spear-pointed drill in this manner cannot be productive of harm unless in such a case as Dr. Ottolengui cites, which is followed by excessive hemorrhage. In a majority of the cases I ligate the tooth in position; I do not use silk or linen ligatures, but prefer the one suggested by Dr. Farrar in retaining teeth—soft annealed brass wire, No. 31. I have used it repeatedly for that purpose, and when implanting, use it for holding the tooth in position. It does not corrode, and is without any irritating effect upon the gum and soft tissues. It has given entire satisfaction, and I have used it almost exclusively. In some cases, when I first practiced implantation, I used silk ligatures; but they were not as satisfactory in any way as the brass wire. During the time of repair and healing, antiseptic measures are kept up by using phenol-sodique as a mouth wash.

*Dr. Ottolengui*—Dr. Kirk comments upon the insertion of the tooth under the existing circumstances. I was advised to take the tooth out, although when I put it in the hemorrhage stopped. The packing had to be put in with considerable solidity, because the blood would not clot without doing so, and when it finally stopped flowing externally, it still oozed into the antrum.

Dr. Kirk may be right when he asserts that there is no trouble to be apprehended in opening into the antrum, but he must remember it is not an ordinary cavity. It is one in which the opening is at the top; fluids cannot escape until it is two-thirds full, when they flow into the nares; it is important to avoid puncturing it, because in the passage you may rupture some vessel that will pour its contents into it. There is a difference in opening into the antrum in disease and drilling into it in implantation. In disease, there is a diseased root that is first removed, a long socket exists and only a little bone is removed. In drilling a socket into the new bone it is different. This not only has arterioles in it, but it is filled with blood, and to rupture a vessel gives rise to a possibility of the blood pouring up into the cavity.

My instruments are a combination of the drill and the reamer. They have nine leaves to them, and only three go to the top. It is very much simpler to use a construction that will not only drill the depth you want, but also do lateral reaming. It makes a difference whether the operation is performed in one hour or in a few minutes.

*Dr. James Truman*—I am sorry it was necessary for Dr. Kirk to leave. He did not seem to think the hemorrhage was a matter of any great importance, that it could have been readily controlled, and that the placing of the tooth was a wrong measure. This may have been true, but what was he to do about a hemorrhage of that character? It was necessary to have some pressure upon that arteriole to stop the hemorrhage and produce coagulation. In that case he would necessarily force blood into the antrum, which would become decomposed. I do not see how it could be possible to keep the cavity open. I do not think we can penetrate the antrum with impunity. If we do so there will be irritation, and inflammation will be set up in the lining membrane and the development of micro-organisms. If there is no harm in opening into the antrum, and no possibility of evil, then I have not learned my lesson well.

*Dr. Ottolengui*—One thing seems to have escaped Dr. Kirk—that the blood came from the lining membrane itself. It would be difficult to get a compress up there to stop the bleeding, even if the character of the blood was such as would readily coagulate.

*Dr. James Truman*—I wish to express my gratification, not only for the paper, but for the kindness of Dr. Ottolengui in presenting the Society with these illustrations, which show very clearly the danger of this operation, with the possibility of trouble, and other facts in connection with it, and I wish to offer a resolution that the thanks of this Society be offered the Doctor, not only for the paper, but for the kindly presentation of the pamphlets.

Subject passed.

Dr. Head then presented some new points in relation to root filling.

*Dr. Jos. Head*—In my paper of last month I asked some questions at the end which were not, to my mind, satisfactorily answered. They were as follows:

Does not gutta-percha leak when used for filling canals?

Do not oxy-chloride of zinc and phosphate of zinc leak when used for filling canals?

If they leak, do they not place the tooth in danger of alveolar abscess?

Do not these leaky substances make a harbor for germs of of putrefaction?

And, lastly, cannot a thoroughly water-tight antiseptic stopping be made with cotton soaked in a 30 per cent solution of carbolized cosmoline?

It was said by Dr. Truman that gutta-percha, when used as a canal dressing, in his opinion, did not leak. My experiment with glass tube tests, which I admit are very crude tests, was criticized on the ground that the glass expanded. It was not denied that oxy-chloride of zinc would absorb moisture. One gentleman, a visitor, advised me to take a glass tube drawn down at the end to the size of the apical foramen of a tooth. This, when filled with gutta-percha, he said, would exclude moisture for months. I did not carry out his experiment, as the unequal expansion of the glass and gutta-percha again could have been brought forward. I concluded to set aside all unnecessary complication by taking natural teeth—three, and opened into the canals and filled them much more thoroughly than it could be possible to do when situated in the mouth. After cleansing the canals by careful drilling into one of the teeth, warm gutta-percha was inserted until it streamed from the apical foramen. The other two teeth were respectively filled with oxy-chloride of zinc and cotton soaked in carbolized cosmoline. The holes in the crowns of these teeth were then filled with gold. Having operated on these teeth January 7th, I dipped them in a solution of analine ink. They were removed on the 1st of February, having been submerged just twenty-four days. I then laid bare the canals, and here they are, gentlemen, for your inspection. I think you will all perceive that the gutta-percha has not only leaked, but even its surface, to a slight degree, has absorbed the ink. The oxy-chloride of zinc is permeated with ink, while the canal guarded by cotton and carbolized cosmoline is absolutely intact.

The cosmoline has also permeated the structure of the dentine, making it water-tight and antiseptic. But the chief merit of this filling lies in the fact that it can be readily removed. It has been said by my friends that I do not fill teeth with cotton, but fill them with cosmoline. And yet, in the same breath, they assert that I sometimes fill teeth with cotton and carbolic acid, objecting to this practice on the ground, that the carbolic acid evaporating, leaves the cotton ungarded.

Why do not they say that I fill teeth with carbolic acid only? It would be just as logical. They must know that the cotton acts as a vehicle for holding an antiseptic. No one, at the present day, thinks of using it unguarded. Cotton, when saturated in cosmoline, renders venting of the canal much more easy of accomplishment than when cosmoline alone is used.

The great advantage cosmoline has over all other medicaments, lies in the fact that it readily permeates the entire structure of the tooth, thus rendering the tooth water-tight.

*Dr. James Truman*—Cosmoline as a filling material may be indestructible ; if you can work it into a canal. I have not the slightest question but that it would make a good filling; and if a few fibres of cotton go in, it will be good. My controversy was in the use of cotton and carbolic acid. I said that carbolic acid would eventually lose its power in the root canal, and that it would leave a very good field for micro-organisms. Dr. Head has taken the precaution to use cosmoline. I have no controversy with that. I do not know from any experiment that it will preserve teeth. I take his word for it, and I believe it is possible to be used; but I would like to have sections made of these teeth which he presents, and examine them under the microscope. To be sure, it looks as if the teeth have leaked; but I would prefer, as a positive test, before deciding the question, that they be made up into sections, and then be carefully shown under the microscope.

*Dr. Head*—Dr. Truman has nothing to say against cosmoline. Then let us lay that aside. He says that he has something to say against cotton soaked in carbolized cosmoline. The specimens which I have just shown to you show that oxy-chloride of zinc and gutta-percha absorb moisture. What more can cotton do? He finds fault with cotton, and yet approves of oxy-chloride and gutta-percha. I should like Dr. Truman to tell me in what way oxy-chloride of zinc and gutta-percha are superior to cotton. Cotton is not only easily removed, but is also capable of holding an antiseptic. Oxy-chloride of zinc and gutta-percha do *not* hold medicaments. But, perhaps, some one of you will say that oxy-chloride of zinc is in itself an antiseptic. I think anyone who has smelt any oxy-chloride filling that has been in the mouth for six months will hold an entirely different opinion. Such a filling, when examined, will be found to contain gas sufficient to make half a dozen alveolar abscesses.

*Dr. Chupein*—I have used carbolic acid or creosote and cotton for thirty odd years, and I have never been able to see that the practice has been detrimental to the salvation of the teeth, the canals of which have been filled in that way. Dr. W. H. Trueman filled canals for a great many years in that way. At a meeting of the Pennsylvania Association of Dental Surgeons, he said he had been using gutta-percha. I asked him if he had gone back on cotton, he said " No, but I have been experimenting in deference to the opinion of others." I have used cosmoline with cotton, but have not had experience enough to say whether it is more favorable.

*Dr. D. N. McQuillen*—For five years I used cotton a great deal in canal filling, and had a number of abscesses. During the past five years, have not used cotton as a permanent filling, and have had a different experience.

*Dr. Head.*—In my paper, I said that it is not sufficient to fill the tip of the canal with cottton, the root should be filled with cotton up to the pulp chamber.

The great objection to gutta-percha lies in the fact that when a tooth is filled with this material, should inflammation arise, the patient is almost to a certainty compelled to undergo the pains of alveolar abscess.

With cotton this difficulty does not arise, as its fibres can be readily removed. When, of course, the tooth being vented, the gas escapes and the patient is relieved.

*Dr. Ottolengui*—We, in Brooklyn, have been converted by Dr. Van Wert to his method of filling root-canals. He uses:—

℞. Iodol .............................. ʒj

Oxide of zinc ...................... ʒij

Cosmoline ........................... q. s.

Make into a stiff paste; it requires dexterity to make it so.

We fill canals with it, and we do not have any abscesses.

*Dr. Head.*—While that makes a good filling, it does not, in my opinion, make a better filling that pure carbolized cosmoline. I did not say that the principal use of cotton was to act as a vehicle. The chief advantage is that the filling can be easily removed, thus saving the patient from alveolar abscess.

### INCIDENTS OF PRACTICE.

*Dr. B. F. Place*—I extracted a number of upper and lower teeth for a lady, about thirty years of age. The lower teeth were badly decayed and diseased. The lady went away without any trouble; the gums healed up nicely; but two months afterwards, she complained of a great deal of pain in the upper and lower jaws. Whenever she exposes herself to a draft of air or goes out, she has great pain along the zygoma, and often there is some slight inflammation, more on the left than on the right side. I was inclined to believe that there might be a small piece of root remaining; but I

was careful in removing them, and know there was no sign of foreign bodies there, except a small piece of the process, which had been imbedded in the gum, and which I removed. She complained of pain, which at times, is better than at others. The soreness is more on the left side than on the right. She has not had any teeth in. I thought possibly that it might be some trouble with the nerves, and I would ask the opinion of the gentlemen here.

*Dr. S. E. Gilbert*—I have the models of a couple of cases of irregularity which I have corrected. The first is that of a young lady of twenty years of age, where the left superior lateral is thrown inside of the arch and overlapped by the central incisor; the inferior teeth closing over its labial aspect. To correct this, I used a "cross-bar," as described and illustrated by Dr. Shaw, in April Cosmos, 1888, page 215. The correction was accomplished in eleven days, causing very little soreness. The retaining fixture consists of a very thin platinum band (made to fit the tooth), with a cross-bar soldered to it, so that it rested against the labial surfaces of the adjoining teeth. This fixture was cemented to the tooth and allowed to remain.

The second case is that of a Miss of fifteen, and as you will see by the cast, the superior incisors are very prominent, so much so, that the lips could not be closed. This was corrected by means of a vulcanite plate, made to fit close to the molars and covering the palatal surface of the mouth. A rubber ring cut from small french tubing was fastened to the centre of the posterior part of the plate, and a silk ligature passed through the ring, the plate placed in the mouth and the ligature drawn forward, passing it between the centrals and tied over a cross-bar of wood. The tension brought to bear by the stretching of the rubber ring soon moved the teeth backward and into place, as shown in cast No.2,which was taken after the case was completed. The improvement in the features is very marked.

Subject passed.

